# Sema3E/Plexin-D1 Mediated Epithelial-to-Mesenchymal Transition in Ovarian Endometrioid Cancer

**DOI:** 10.1371/journal.pone.0019396

**Published:** 2011-04-29

**Authors:** Chun-Hsien Tseng, Karl D. Murray, Mu-Fan Jou, Su-Ming Hsu, Hwai-Jong Cheng, Pei-Hsin Huang

**Affiliations:** 1 Graduate Institute of Pathology, College of Medicine, National Taiwan University, Taipei, Taiwan; 2 Center for Neuroscience, University of California Davis, Davis, California, United States of America; 3 Department of Pathology, National Taiwan University Hospital, Taipei, Taiwan; Institut de Génomique Fonctionnelle de Lyon, France

## Abstract

Cancer cells often employ developmental cues for advantageous growth and metastasis. Here, we report that an axon guidance molecule, Sema3E, is highly expressed in human high-grade ovarian endometrioid carcinoma, but not low-grade or other ovarian epithelial tumors, and facilitates tumor progression. Unlike its known angiogenic activity, Sema3E acted through Plexin-D1 receptors to augment cell migratory ability and concomitant epithelial-to-mesenchymal transition (EMT). Sema3E-induced EMT in ovarian endometrioid cancer cells was dependent on nuclear localization of Snail1 through activation of phosphatidylinositol-3-kinase and ERK/MAPK. RNAi-mediated knockdown of Sema3E, Plexin-D1 or Snail1 in Sema3E-expressing tumor cells resulted in compromised cell motility, concurrent reversion of EMT and diminished nuclear localization of Snail1. By contrast, forced retention of Snail1 within the nucleus of Sema3E-negative tumor cells induced EMT and enhanced cell motility. These results show that in addition to the angiogenic effects of Sema3E on tumor vascular endothelium, an EMT strategy could be exploited by Sema3E/Plexin-D1 signaling in tumor cells to promote cellular invasion/migration.

## Introduction

Malignant progression of a tumor often involves acquisition of enhanced migratory ability in cancer cells for local invasion and distant metastasis, both of which are the main determinants for clinical morbidity and mortality. Similar biological processes occur throughout normal embryonic development, as well as in certain physiological conditions such as wound healing [1], [2]. Insight from developmental biology could therefore help us understand the invasive nature of malignant progression of a tumor.

Semaphorins (Sema) are a large family of secreted and membrane-associated proteins that provide environmental cues to mediate diverse developmental processes including neuronal cell migration, axon guidance, vasculogenesis, branching morphogenesis, and cardiac organogenesis [3]–[8]. Semaphorins bind plexin and/or neuropilin receptors to transduce intracellular signals. At present, five classes of semaphorins, two neuropilins and four families of plexins are identified in mammals [6]. Recent evidence also suggests semaphorin/plexin signaling is involved in tumorigenesis [9]–[11]. However, their roles are quite diverse and depend on the specific tumor context and the composition of semaphorins, plexins, and their intracellular signal responsive elements. Semaphorin/plexin signaling can either promote or inhibit tumor growth by directly regulating cell migration or cell apoptosis. Semaphorin/plexin signaling can also indirectly control tumor invasive growth through regulation of angiogenesis or tumor immunity [10], [12]–[16].

**Figure 1:**
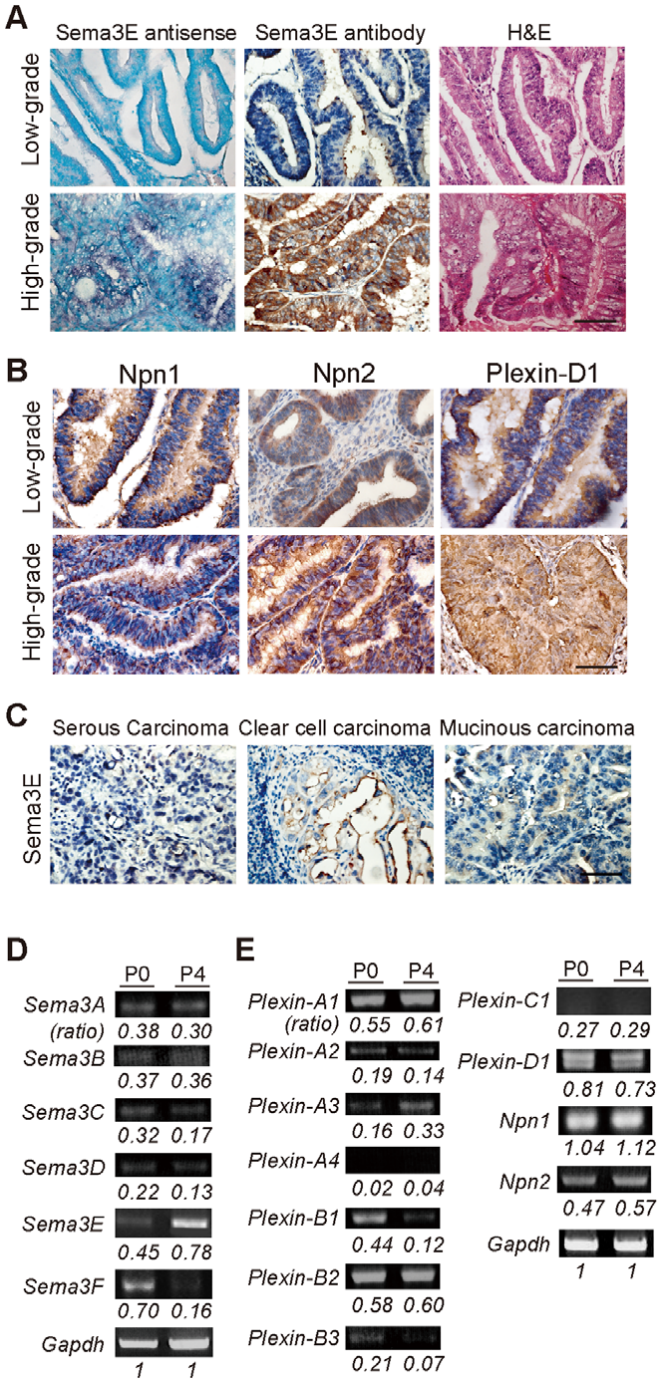
Differential expression of class 3 semaphorins, neuropilins, and plexins in human ovarian epithelial cancers. **A**, **B.** RNA *in situ* hybridization with antisense *Sema3E* probe and immunohistochemistry with a polyclonal antibody against Sema3E show differential expression of mRNA and protein respectively in cells throughout tissue sections from high and low grade human ovarian epithelial cancers. Hematoxylin and eosin staining (H&E) was performed on adjacent sections to indicate general tissue cytoarchitecture. Scale bar, 20 µm. **C.** Immunohistochemistry shows a lack of Sema3E expression in three different non-ovarian epithelial carcinomas. **D**, **E.** Semi-quantitative RT-PCR analysis of class 3 semaphorin, neuropilin and plexin expression in P0 and P4 OEC cells shows variable levels of expression. All density values for PCR fragments are normalized to that of *Gapdh* for quantification.

In a screen of class 3 semaphorins in tumor tissue arrays (some examples are shown in Fig. S1C), we identified Sema3E as specifically expressed in high-grade ovarian endometrioid carcinoma, a subtype of epithelial ovarian cancers (Fig. 1). Clinically, most diagnoses of high-grade ovarian cancer have poor-prognosis with tumor metastasis and are refractory to chemotherapy underscoring the need to thoroughly understand the pathogenesis of epithelial ovarian cancers and their progression [17]. Using a human ovarian endometrioid carcinoma cell line and derived sublines with different invasive/migratory capabilities [18], we investigated the interrelation of Sema3E molecular and cellular signaling mechanisms and tumor invasiveness. We report here that Sema3E from tumor cells can act on themselves through Plexin-D1 to induce EMT and concomitantly facilitate cell migration and malignant progression.

## Results

### Sema3E is over-expressed in high-grade ovarian endometrioid carcinoma

Based on preliminary immuno-screening results, we investigated in detail the expression of Sema3E and its receptors, Plexin-D1 and Neuropilin-1 (Npn1), in human ovarian endometrioid carcinomas. Tumor samples were obtained from 40 patients diagnosed as primary ovarian endometrioid carcinoma at National Taiwan University Hospital from 1995–2002. In addition to the primary tumor, 9 cases had lymph node metastasis, and matching pairs of primary tumor and metastatic iliac lymph node were available in 7 cases. Among these 40 tumor samples, 25 were diagnosed as high-grade ovarian endometrioid carcinoma, and in all cases a significant level of Sema3E protein and transcript was detected. Sema3E expression was independent of patients' age and tumor stage, but was significantly correlated with tumor grade (Table 1, and Fig. 1A, bottom panels). By contrast, most cases with low-grade ovarian endometrioid carcinoma exhibited barely detectable Sema3E levels (Fig. 1A, top panels). Plexin-D1 and Npn1, components of the Sema3E receptor, showed similar degrees of immuno-labeling in low-grade and high-grade tumors (Fig. 1B). Moreover, Sema3E was specifically over-expressed in high-grade ovarian endometrioid carcinoma in that other types of ovarian epithelial tumors including serous, mucinous and clear cell tumors, low-grade and high-grade alike, did not express Sema3E (Fig. 1C). These data indicate a strong correlation between Sema3E expression and high-grade human ovarian endometrioid carcinoma.

**Table 1:** Sema3E Expression Correlates Positively with High Grade Ovarian Endometrioid Carcinoma.

	Sema3E			
	Negative	Positive	Total	*P* value*
**Age**				
<55 y/o	7 (25%)	21 (75%)	28	0.589
>55 y/o	4 (33.3%)	8 (66.7%)	12	-
**Stage**				
Low (I, II)	6 (33.3)	12 (66.7%)	18	0.455
High (III, IV)	5 (22.7%)	17 (77.3%)	22	-
**Tumor grade**				
Low	11 (84.6%)	2 (15.4%)	13	1.98218E-08
High	0 (0%)	27 (100%)	27	-
**Lymph node metastasis**				
Negative	-	-	33	-
Positive	3 (42.8%)	4 (57.2%)	7	0.322

*Chi-square test.

We then examined the expression of semaphorins and their receptors in human ovarian endometrioid carcinoma cell lines. A human ovarian endometrioid carcinoma (OEC) cell line designated as P0 was previously established from a 63-year-old female patient with stage IIIc endometrioid carcinoma. A subline designated as P4 was also established by selecting clones that exhibited enhanced migratory/invasive ability assayed by transwell chamber and wound migration [18]. Contrary to the differential migratory/invasive capacities of P0 and P4 cells, no apparent difference in cell proliferation and apoptosis was observed [18] (also see Fig. S2C, D, and E). Semi-quantitative RT-PCR analyses by gel electrophoresis (Fig. 1D) or in real-time (Fig. S2B) showed that among class 3 semaphorins levels of Sema3E were significantly higher (>10-fold) in high-invasive/migratory P4 cells compared with P0 cells. By contrast, Sema3F generally regarded a tumor suppressor was down-regulated in P4 cells [10], [19]. Although variable plexin and neuropilin expression was observed, Plexin-D1 and Npn 1 were equally expressed in the low-invasive P0 and high-invasive P4 cells (Figs. 1E and S2B). RNA *in situ* hybridization analysis on cultured single ovarian endometrioid cancer cells confirmed these results (Fig. S2A). This suggests Sema3E expression in ovarian endometrioid cancer cells could be involved in the acquisition of cellular invasive/migratory ability.

### The p61-Sema3E isoform is likely to act in an autocrine manner through Plexin-D1 to enhance the invasive/migratory ability of ovarian endometrioid cancer cells *in vitro* in a concentration-dependent manner

**Figure 2:**
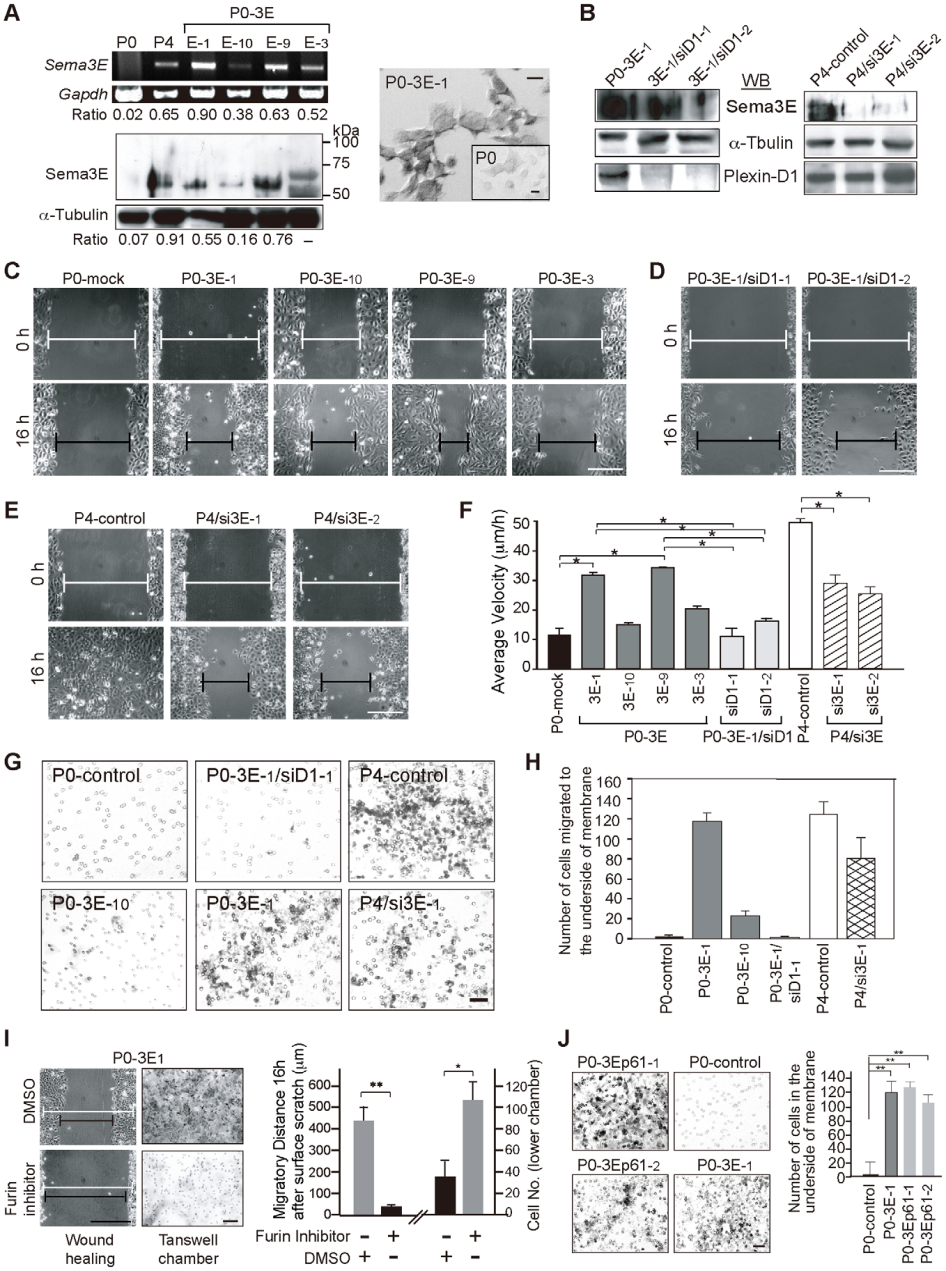
Sema3E mediates the invasive/migratory ability of OEC cells *in vitro* in a Sema3E-p61/Plexin-D1 dose-dependent manner. **A.** Modified P0 cell lines (P0-3E-_x_) express variable levels of Sema3E as measured by semi-quantitative RT-PCR analysis (left top) and Western blotting (left bottom). Expression values were normalized to *Gapdh* and œ-Tubulin for mRNA and protein, respectively. RNA *in situ* hybridization probes by Sema3E in P0-3E-_1_ compared with P0 cells (right). **B.** Western blotting of Sema3E and Plexin-D1 in P4/si3E clones (RNAi-1, -2 for targeting two different sequences of Sema3E) and P0-3E-_1_/siD1 clones (RNAi-1, -2 for targeting two different sequences of Plexin-D1). **C**, **D**, **E**, **F.** Brightfield photomicrographs from wound-healing migration assays of various P0-3E (C), P0-3E-_1_/siD1 (D), and P4/si3E (E) cell lines at the beginning (0 h) and 16 hours after surface scratch. Wound edges were marked with white lines (0 h) or black lines (16 h). Scale bar, 0.5 mm. A summary of the average migrating velocity (µm/h) for each clone is plotted (F) with standard deviation (n≥12). *, *P*<0.05, paired *t*-test. **G**, **H.** Representative images (G) and graph summary (H) of the transwell chamber invasion assay (n≥6). Scale bar, 30 µm. **I.** The furin inhibitor, decanoyl-RVKR-chloromethylketone, alters migratory ability of P0-3E-_1_ cells in wound-healing and transwell chamber migration assays. DMSO was used as a control. White line, 0 h in wound-healing process; black line, 16 h after surface scratch. **, *P*<0.005, *, *P*<0.05, paired *t*-test, n = 5. Scale bar, 0.5 mm. **J.** Transwell chamber invasion assay for P0 cells stably expressing p61-Sema3E isoform (P0-3Ep61-_1_ and P0-3Ep61-_2_) compared with P0-3E-_1_ cells and P0 cells. **, *P*<0.005, paired *t*-test, n = 4. Scale bar, 0.3 mm.

To investigate the role of Sema3E in mediating invasiveness and/or migratory ability during tumor progression, we performed gain and loss of function studies in OEC cells. First, we over-expressed Sema3E in parental Sema3E-negative, low-invasive P0 cells. These stable cell lines (designated P0-3E-_1_, P0-3E-_3_ and P0-3E-_10_) exhibited variable degrees of Sema3E expression as measured by semi-quantitative RT-PCR, immunoblotting, and RNA *in situ* hybridization (Fig. 2A). Second, Sema3E expression in highly invasive P4 cells was stably knocked down by RNAi-mediated depletion. We obtained two clones, designated P4/si3E-_1_ and P4/si3E-_2_ targeted by different RNAi sequences that showed 75%∼90% decrease in Sema3E compared to parental P4 cells (Fig. 2B, right panels). Control OEC clones were transfected either with empty-vector (-mock) or scrambled RNAi sequences (-control). Flow cytometry analysis and trypan blue exclusion experiments indicated that cell viability and proliferation were not altered in P0-3E and P4/si3E clones (Fig. S2C and D).

The invasive/migratory abilities of OEC clones were evaluated by *in vitro* wound-healing, 3-dimensional (3D)-matrigel migration, and transwell chamber invasion assays. In the wound-healing assay, P4-mock and P0-3E-_1_ cells started to fill the wound as early as 4 hours after the scratch, whereas P0-mock cells exhibited slow motility even after 16 hours. The P0-3E clones migrated in a Sema3E dose-dependent manner such that the P0-3E-_1_ and P0-3E-_9_ cells, expressing relatively high amounts of Sema3E, moved faster and farther than the P0-3E-_3_ and P0-3E-_10_ cells, which expressed lower amounts of Sema3E (Fig. 2C and F). Conversely, depletion of Sema3E in P4 cells slowed down the migratory velocity and distance (Fig. 2E and F). Similar changes were observed in the matrigel migration assay and the transwell chamber invasion assay: P4 and P0-3E-_1_ cells exhibited a significantly greater invasive potential compared with P0-3E-_10_, P4/si3E or parental P0 cells where Sema3E levels are reduced or absent (Figs. 2G, H and S2E). Therefore, over-expression of Sema3E in low-invasive OEC cells enhances cellular migratory and invasive abilities *in vitro* in a does-dependent manner, and knockdown of Sema3E diminishes the migratory/invasive abilities in high-invasive OEC cells.

Sema3E binds directly with Plexin-D1 to transduce intracellular signal during embryonic or tumor angiogenesis [20]. We determined whether Plexin-D1 in OEC cells is required for Sema3E mediated migratory/invasive ability that is unrelated to angiogenesis. Two stable knockdown clones, P0-3E/siD1-_1_ and P0-3E/siD1-_2_, were generated by RNAi targeting of separate Plexin-D1 sequences which reduced Plexin-D1 levels by 85%–95% as determined by western blotting (Fig. 2B, left panels). Knockdown of Plexin-D1 affected neither the expression of Sema3E, nor the viability or proliferation of cells (Figs. 2B, S2C and S2D). However, the invasive/migratory phenotype conferred by over-expression of Sema3E was reversed by loss of Plexin-D1 (Fig. 2D, F, G, H). These results suggest that Sema3E confers a migratory ability to OEC cells through Plexin-D1 in an autocrine manner.

**Figure 3:**
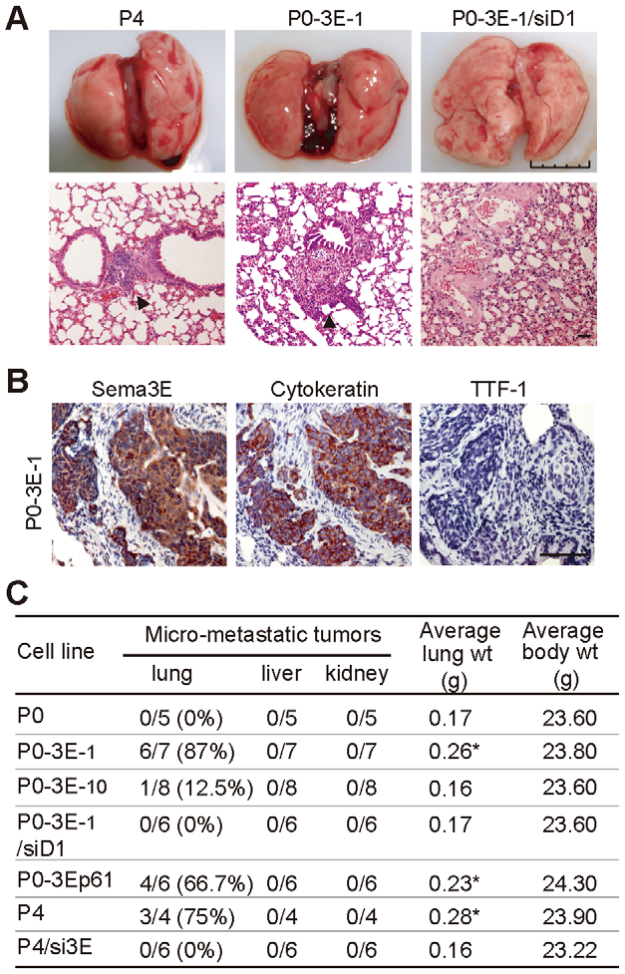
Sema3E/Plexin-D1 signaling mediates metastatic growth of ovarian endometrioid cancer cells *in vivo*. **A.** Gross and microscopic changes in lungs after intravenous (i.v.) injection into NOD/SCID mice of OEC cell lines expressing different Sema3E/Plexin-D1 activities. Arrowhead indicates microscopic tumor nests near the vascular tree. Scale bar, 50 µm. **B.** Immunohistochemistry of Sema3E, cytokeratin, and TTF1 in pulmonary metastatic tumors of NOD/SCID mice i.v. injected with P0-3E-_1_ cells. **C.** Summary of *in vivo* metastatic experimental results. The number of mice with micro-metastatic tumorous lesion of the lung (left to the slash) versus the total number of mice examined (right to the slash) is shown. Average lung weight differed between groups (*, *P*<0.05, Student's *t*-test).

Full-length class 3 semaphorin proteins are subject to convertase-mediated cleavage, thereby generating multiple isoforms with differential activities [15], [21]. In breast cancer cells, p87-Sema3E protein is cleaved by furin into p61 and p26 isoforms, and it is p61-Sema3E that induces angiogenesis for invasive growth [15]. When performing Sema3E immunoblotting in the Sema3E-expressing OEC cells, we noticed that a p61-sized band instead of the full-length p87-Sema3E was detected, even though all P0-3E clones were transfected with a full-length Sema3E cDNA construct (Fig. 2A). We examined whether this p61-protein corresponded to furin-processed Sema3E. Immunoblotting showed that furin was present in all OEC cells (Fig. S3A). When furin activity was inhibited by decanoyl-RVKR-chloromethylketone, expression of p61-Sema3E was significantly reduced while full-sized p87-Sema3E became evident (Fig. S3B). Moreover, Sema3E-mediated migratory/invasive activities were compromised in the presence of furin inhibitor (Fig. 2I). To examine the possibility that p61-Sema3E alone is sufficient for promoting the migratory/invasive ability of OEC cells conferred by Sema3E, we established P0-3Ep61 clones that expressed only the p61-Sema3E isoform. Both *in vitro* (Figs. 2J, S3C and S3D) and *in vivo* (Fig. 3C) studies demonstrated that expression of p61-Sema3E alone was sufficient to promote migratory/invasive ability in OEC cells. RNAi-mediated knockdown of Plexin-D1 in OEC cells stably expressed p61-Sema3E diminished migratory ability conferred by p61-Sema3E (data not shown). For unknown reasons, we could not generate OEC cells stably expressing mutant p87-Sema3E resistant to furin cleavage. Therefore, we tested conditioned media from COS cells transiently transfected with either p61-Sema3E or p87-Sema3E on cellular motility of Sema3E-negative/Plexin-D1-positive P0 cells. Indeed, exogenous p61-Sema3E-conditioned medium dose-dependently promoted cell migration and this effect was weakened when Sema3E was depleted from the medium by pre-incubation with anti-Sema3E antibody (Fig. S3E and F). When unprocessed p87-Sema3E-conditioned medium was applied, no enhancement in cell migration was observed (data not shown). Collectively, these data indicate that p61-Sema3E, but not full-length p87-Sema3E, promotes a Plexin-D1 dependent migratory ability in OEC cells.

### Sema3E/Plexin-D1 signaling promotes metastasis of ovarian endometrioid cancer *in vivo*

We next evaluated the *in vivo* metastatic capacity of OEC cells with different Sema3E/Plexin-D1 activity in NOD/SCID mice. Intravenous injection of Sema3E-negative P0 cells at no time resulted in tumor metastasis in any organ examined at 8 weeks after cell intravenous injection. By contrast, injection of Sema3E-expressing OEC clones caused metastatic tumor growth in the lung as evidenced by an increase of total lung weight (Fig. 3C), gross intrapulmonary hemorrhage (Fig. 3A, top panels), and the presence of microscopic tumor nests near the vascular trees (Fig. 3A, bottom panels). The pulmonary tumors originated from the injected OEC cells as demonstrated by positive immunoreactivity for human Sema3E and cytokeratin (markers for human cells), but not TTF-1, a marker for pneumocytes (Fig. 3B). In addition, a Sema3E dose-dependent and Plexin-D1-dependent metastatic effect was observed. A higher percentage of pulmonary micro-metastases developed in mice injected with OEC cells expressing high levels of Sema3E (P0-3E-_1_, P0-3Ep61, P4 cells), while cells with low Sema3E/Plexin-D1 activity (P0-3E-_10_, P4/si3E, P0-3E-_1_/siD1) had a lower percentage of tumors (Fig. 3C). Also, p61-Sema3E alone was able to confer pulmonary micro-metastasis on parental P0 cells *in vivo* (Fig. 3C). All together, these results demonstrate that p61-Sema3E through Plexin-D1 signaling is responsible for conferring a dose-dependent stimulation of pulmonary metastatic growth of OEC cells.

### Sema3E/Plexin-D1 signaling alters the dynamic of single OEC cell morphology, consistent with a change in cell motility

**Figure 4:**
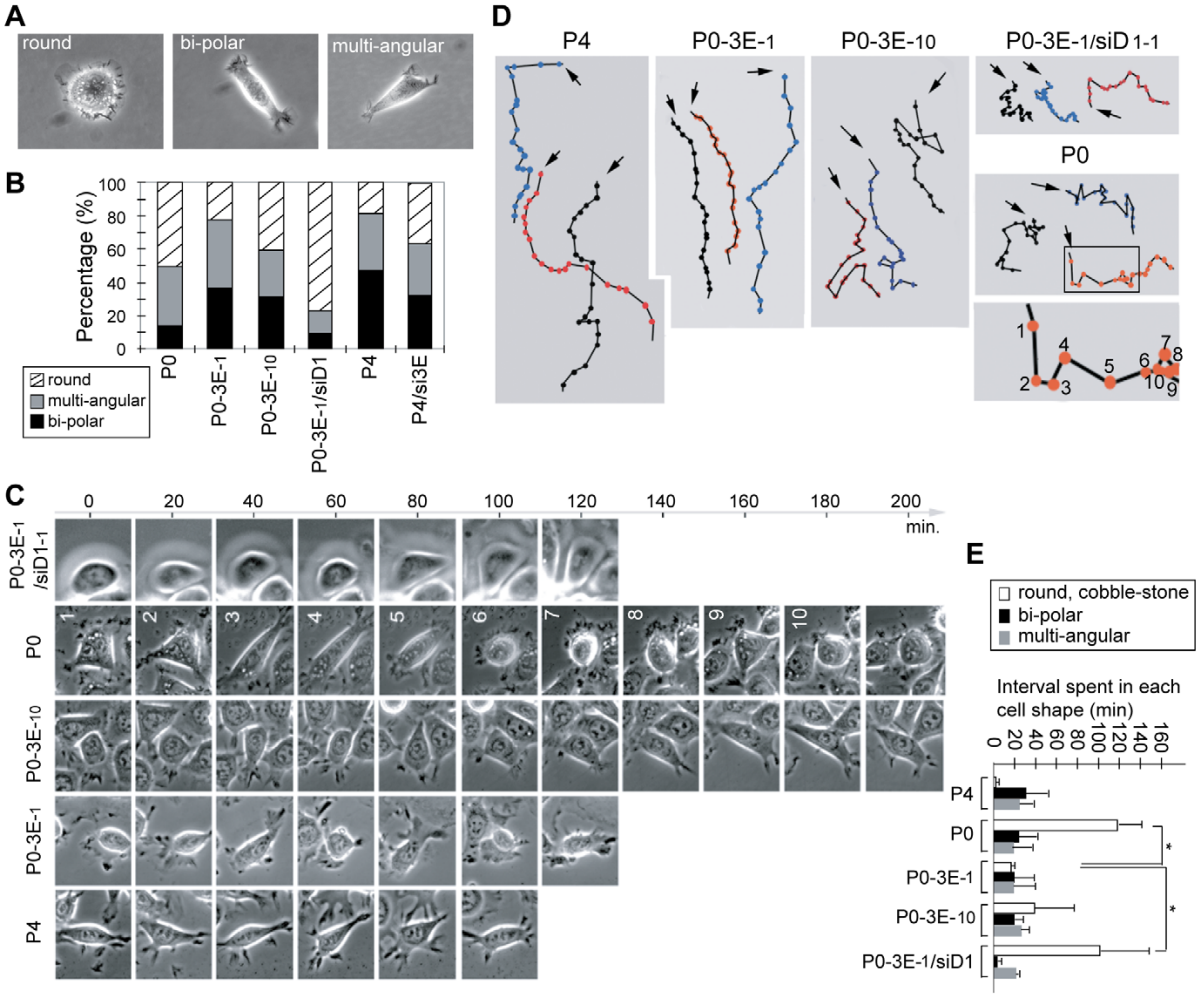
Sema3E/Plexin-D1 activity alters the cytomorphology of ovarian endometrioid cancer cells. **A.** Representative images of three distinct cell shapes of OEC cells as round, bi-polar and multi-angular. **B.** Quantification of the percentage of each morphology in an overall population (n≥400) of OEC clones with variable activity of Sema3E/Plexin-D1. **C**, **D**, **E.** Time-lapse tracing (up to 200 minutes with 20-min tracing intervals) of the morphological dynamics of single OEC cells in a wound-healing process (C). Single OEC migratory paths were plotted (D). Arrows indicate the start of each tracing, and colored dots indicate the position of the traced cell at each time point. Each step in the migratory sequence was labeled with the numbers same as those in (C). The bar graph in (E) summarized average time-interval spent by each OEC cell on each distinct cell shape. *, *P*<0.05, paired *t*-test.

We observed changes in OEC cellular morphology that correlated with the activity of Sema3E/Plexin-D1 signaling. Cells with high Sema3E expression and rapid cell motility such as P0-3E-_1_ and P4 cells were usually spindle-shaped with a bi-polar or multi-angular fibroblastoid appearance (Fig. 4A and B), while P0, P0-3E-_10_, P4/si3E, and P0-3E-_1_/siD1 cells which had low Sema3E/Plexin-D1 activity and low migratory ability, maintained a higher percentage of round shapes with a cobblestone-like appearance (Fig. 4A and B). We examined this correlation in detail, by tracing individual OEC cell migration routes and performing time-lapse photography during the wound-healing assay. In contrast to impressions generated from steady-state images, time-lapse photography revealed a dynamic pattern of cytomorphological cycling between round, bipolar and multiangular shapes that was observed for all OEC cells examined (Fig. 4C). We found that Sema3E/Plexin-D1 activity significantly influenced the time OEC cells were observed in a round shape. Thus, P0 and P0-3E-_1_/siD1 maintained a round morphology for longer than P4 and P0-3E-1 cells which rapidly transitioned through round shape and progressed quickly into a polarized spindle shape (Fig. 4C and E). Furthermore, single cell tracing revealed that OEC cells with Sema3E/Plexin-D1 signaling tended to keep moving in one direction for a long distance, but cells with low-Sema3E/Plexin-D1 activity frequently paused and changed their migratory path (Fig. 4D). As a consequence, P4 and P0-3E-_1_ cells could move quickly in one direction for a long distance, whereas P0 and P0-3E-_1_/siD1 cells stalled, constantly changed directions, and were unable to move far in one direction (Fig. 4D). This correlates well with the overall migratory abilities of different Seam3E-expressing OEC cells in the wound-healing assay. More intriguingly, the cytomorphological transition from round to spindle shape that is highly associated with the acquisition of cell motility resembles the phenomenon of so-called epithelial-to-mesenchymal transition (EMT) [22], suggesting that Sema3E/Plexin-D1 signaling in OEC cells could be involved in the EMT process.

### Sema3E/Plexin-D1 signals through PI3K and MAPK to induce nuclear Snail1 translocation and epithelial-to-mesenchymal transition

**Figure 5:**
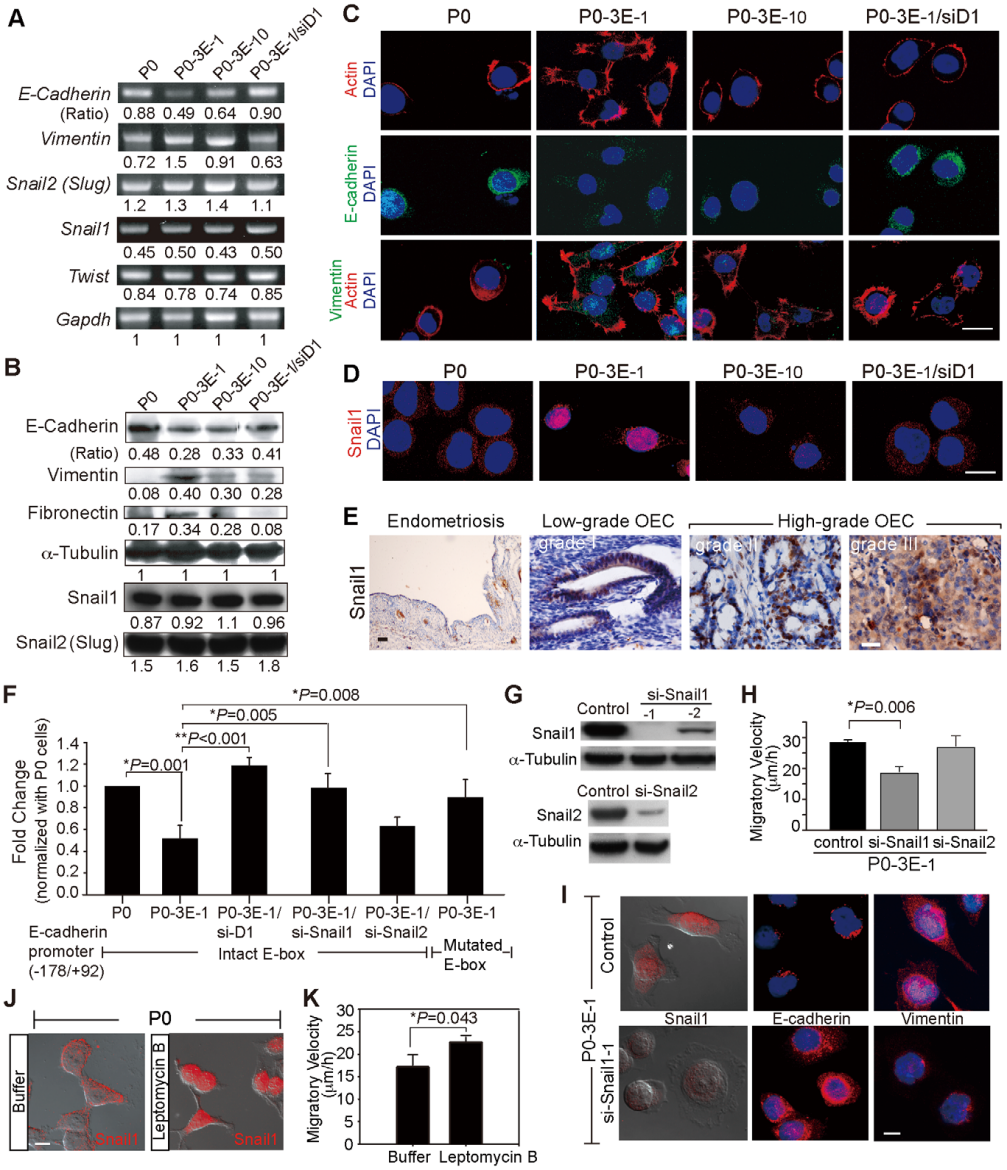
Sema3E/Plexin-D1 mediated epithelial-to-mesenchymal transition depends on nuclear translocation of Snail1. **A**, **B.** Expression of cellular markers of EMT correlates with levels of Sema3E/Plexin-D1 activity in modified OEC cells as measured by semi-quantitative RT-PCR (A) and Western blotting (B). By contrast, members of the Snail family transcription factors that regulate developmental EMT are uniformly expressed in modified OEC cell lines. Signal intensities were normalized to *Gapdh* (A) or œ-Tubulin (B), respectively, and are indicated as ratios. **C**, **D.** Confocal microscopy of OEC cells shows a shift from epithelial (E-cadherin) to mesenchymal (Vimentin) marker expression and a distinct nuclear translocation of Snail1 in modified OEC cells exhibiting greater Sema3E/Plexin-D1 activity. Immunoreactivity for filamentous actin, and nuclear DAPI staining were used as general markers of cell morphology. Scale bar, 20 µm. **E.** Nuclear localization of Snail1 is associated with high grade of human OEC tissue, but not low-grade human OEC tissues. Snail1 was not detected in ovarian endometriosis. Scale bar, 10 µm. **F.** E-cadherin promoter activity, measured by normalized luciferase expression in modified OEC lines, is down-regulated in cells with high Sema3E/Plexin-D1 signaling. This is dependent on Snail1 translocation since knockdown of Snail1 or a mutated E-box prevents transcriptional repression. Error bars show s.e.m., n = 9. **, *P*<0.01, *, *P*<0.05, paired *t*-test. **G.** Western blots illustrate RNAi-mediated knockdown of Snail1 in Sema-3E expressing P0-3E-_1_ cells via lenti-viral infection. **H.** Knockdown of Snail1 expression in P0-3E-_1_ cells decreases migration in wound healing assay. **I.** Knockdown of Snail1 in P0-3E-_1_ cells induces a shift from spindle shaped, vimentin positive to a rounded, E-cadherin positive cellular morphology. Scale bar, 10 µm. **J**, **K.** Pharmacologically-induced retention of nuclear Snail1 by leptomycin B (20 ng/ml) increases migratory ability and induces a spindle shaped cellular morphology in P0 cells. Scale bar, 10 µm.

During EMT, cells often lose their epithelial molecular markers and acquire markers of mesenchymal cells [23]–[26]. We thus examined the molecular profile of different OEC clones to determine whether Sema3E-induced morphological changes were associated with an EMT event. E-cadherin, an epithelial marker, was down-regulated in Sema3E-expressing OEC cells in a Sema3E-concentration-dependent manner, whereas mesenchymal markers vimentin and fibronectin were upregulated (Fig. 5A, B, and data not shown). By contrast, RNAi-knockdown of Plexin-D1 in Sema3E-expressing cells abolished Sema3E-induced molecular changes characteristic of EMT (Fig. 5A and C). Similar Sema3E-associated changes of EMT markers were observed when cells were examined by confocal immuno-fluorescent microscopy (Figs. 5C and S4A). In addition, immunostaining for filamentous actin in Sema3E-expressing cells identified filopodia and lamellipodia formation typical of EMT-induced cytoskeletal changes, but this was not observed in cells with low Sema3E/Plexin-D1 expression (Fig. 5C, top panels).

A major regulator of EMT during embryonic development and tumor progression is the Snail family of zinc finger transcription factors, of which Snail (Snail1) and Slug (Snail2) are the most intensively studied [27]–[29]. Many epithelial cancer cells induce EMT via up-regulation of Snail1 mediated transcription [22]. However, Sema3E expression in OEC cells did not alter mRNA or protein levels of Snail1 (or Snail2 and Twist) (Fig. 5A and B). The function of Snail1 could alternatively be modulated by its subcellular localization so that nuclear translocation of cytosolic Snail1 is required for accessibility of target promoters [30], [31]. We examined whether Sema3E/Plexin-D1 signal modulated the subcellular localization of Snail1 in OEC cells. Immuno-fluorescent microscopy revealed a mixed population of OEC cells with Snail1 either predominantly located in the nucleus, or in the cytoplasm. However, nuclear localization of Snail1 correlated positively with Sema3E/Plexin-D1 activity. In cells with high Sema3E/Plexin-D1 activity such as P0-3E-_1_ cells, most exhibited predominant nuclear localization of Snail1 (64%±8% [mean ± standard deviation of three experiments]), whereas most cells with low Sema3E/Plexin-D1 activity displayed a predominantly cytosolic distribution of Snail1 (for example: P0, 78%±6% cytosolic Snail1 localization) (Fig. 5D). In addition, immunohistochemistry of Snail1 in human ovarian endometrioid carcinomas revealed that high-grade tumors had a higher percentage of cancer cells with nuclear Snail1 and low-grade tumors had predominantly cytosolic Snail1 localization (Fig. 5E). By contrast, no Snail1 expression was observed in endometriosis cyst-lining cells (Fig. 5E). These observations suggest that Sema3E/Plexin-D1 signaling in ovarian endometrioid cancer cells may induce EMT through nuclear translocation of Snail1.

To determine whether Snail1 is a downstream responsive element for Sema3E/Plexin-D1 signaling in OEC cells within the context of EMT and cell motility, we tested whether the transcriptional down-regulation of the epithelial cell marker E-cadherin in response to Sema3E/Plexin-D1 signaling depends on Snail1. As shown in Fig. 5F, luciferase promoter activity assay showed that cells with high Sema3E/Plexin-D1 activity exhibited repressed promoter activity of E-cadherin (for example, compare P0-3E-_1_ cells with P03E/siD1 or P0 cells). By contrast, mutation in E-box (a target recognized by Snail1) or RNAi-knockdown of Snail1 resulted in loss of repression in E-cadherin promoter activity conferred by Sema3E/Plexin-D1. In addition, knockdown of Snail2 in P0-3E-_1_ cells could not disinhibit the repressed promoter activity of E-cadherin (Fig. 5F). We also found that RNAi-knockdown of Snail1 in Sema3E-expressing cells caused the spindle-shaped cells tansformed into rounder shape and the expressions of E-cadherin and Vimentin concomitantly reverted (Fig. 5G and I), indicating that Snail1 is required for Sema3E-induced EMT. In addition, the enhanced migratory ability conferred by Sema3E in P0-3E-_1_ cells was diminished when Snail1 was RNAi-depleted but unaltered by RNAi-depletion of Snail2 (Fig. 5H). On the contrary, forced retention of Snail1 within the nucleus by leptomycin B treatment in Sema3E-negative P0 cells increased cell migratory ability and resulted in transformation into spindle-shaped cells (Fig. 5J and K). This suggests that Snail1 translocation into the nucleus functions downstream of Sema3E signaling to mediate cell morphology and motility. Time-lapse tracing of Snail1 subcellular localization versus cell morphology and migratory velocity revealed a correlation between Snail1 nuclear localization and spindle cell shape as well as with increased migratory velocity (Fig. S4B, C and D). Collectively, these studies indicate that Sema3E/Plexin-D1 signaling in ovarian endometrioid cancer cells could regulate Snail1 translocation to the nucleus in order to induce EMT and concomitantly augment cell motility.

**Figure 6:**
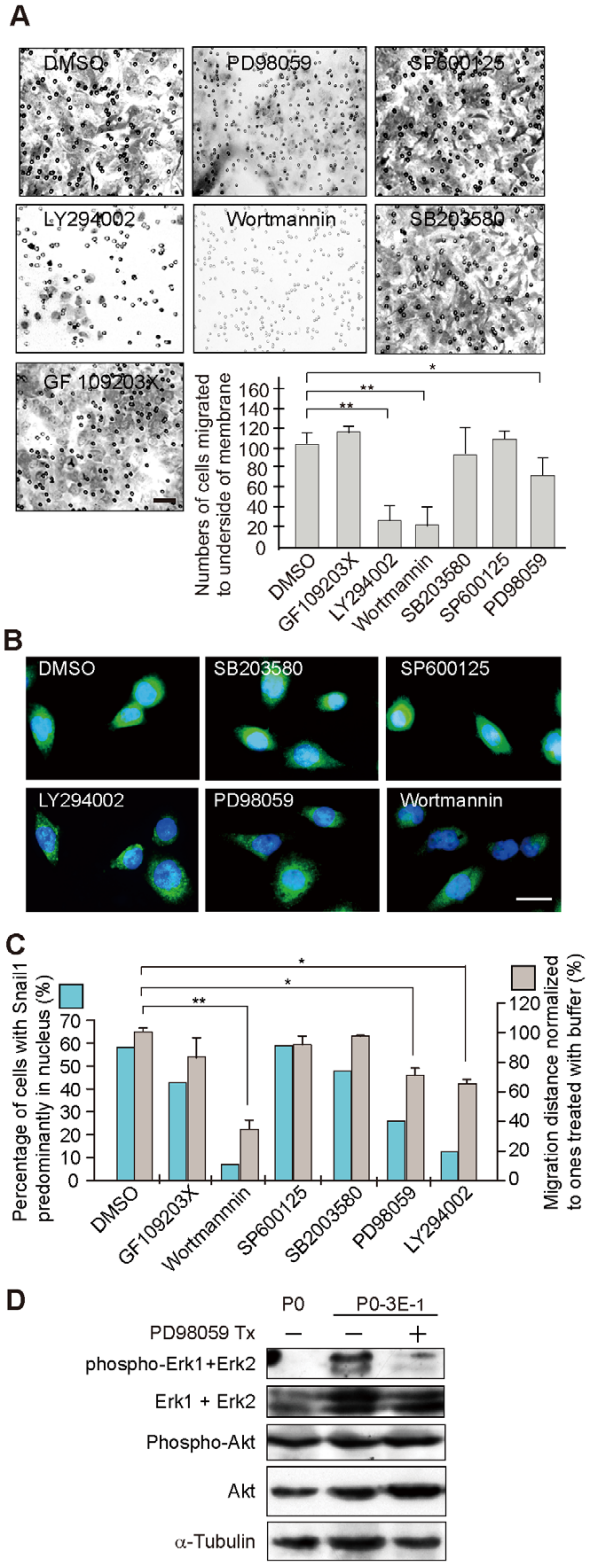
PI3K and ERK/MAPK signaling pathways mediate regulation of Sema3E/Plexin-D1-induced EMT. **A**, Cell transwell invasion assay of P0-3E-_1_ cells reveals a significant decrease in migratory cell activity following pharmacological inhibition of PI3 kinase (LY294002, or wortmannin) and ERK/MAPK (PD98059) but not p38 mitogen activated protein kinase (SB203580), PKC (GF109203X), or Jun-N-terminal kinase (SP600125). Scale bar, 30 µm. **, *P*<0.01, *, *P*<0.05, paired *t*-test. **B.** Confocal microscopy indicates that inhibitors of PI3 kinase and ERK/MAPK specifically prevent nuclear translocation of Snail1 in P0-3E-_1_ cells. Vehicle alone (DMSO) had no obvious effect on Snail1 translocation. Scale bar, 20 µm. **C.** Quantitative analysis of Snail1 nuclear translocation and normalized cellular migration in wound healing assays following pharmacological block of intracellular signaling pathways. **, *P*<0.01, *, *P*<0.05, paired *t*-test. **D.** Increased phosphorylated ERK is associated with increased Sema3E/Plexin-D1 activity in P0-3E_−1_ cells compared with the parental P0 cell line. Inhibition of ERK/MAPK signaling with PD98059 prevented ERK phosphorylation but did not affect overall ERK expression levels or activation or levels of Akt.

The intracellular signaling pathway(s) that might regulate Sema3E/Plexin-D1 mediated translocation of Snail1 and subsequent EMT in OEC cells was investigated by testing the effect of several well-known kinase inhibitors on cell motility and Snail1 subcellular localization. Inhibition of p38 mitogen-activated protein kinase (SB203580), protein kinase C (GF109203X), and Jun N-terminal kinase (SP600125) in Sema3E-expressing OEC cells had no effect on Sema3E-augmented cell migration or on Snail1 nuclear localization. By contrast, inhibition of phosphatidylinositol-3-kinase (PI3K) (LY294002 and wortmannin) or extracellular signal-regulated kinase (ERK)/mitogen activated protein kinase (MAPK) (PD98059) activity resulted in cytosolic retention of Snail1 and a concomitant decrease in migratory ability conferred by Sema3E (Fig. 6A, B and C). Immuno-blotting also demonstrated that Sema3E expression correlated with phosphorylated ERK1/ERK2 expression, which diminished upon PD98059 treatment (Fig. 6D). These studies suggest that activated PI3K and ERK/MAPK are the main intracellular signaling components transducing Sema3E/Plexin-D1 mediated Snail1 nuclear translocation.

## Discussion

Semaphorin/plexin signaling has been broadly linked with tumor growth and metastasis. Here, we demonstrated a strong association between Sema3E expression and high-grade human ovarian endometrioid carcinoma. Moreover, we uncovered an unexpected functional link between Sema3E/Plexin-D1 signaling and epithelial-to-mesenchymal transition, and provided a mechanistic explanation for the augmented cell motility conferred by Sema3E from high-grade tumors. Although a similar pro-invasive effect has been attributed to autocrine Sema3E/Plexin-D1 signaling in colon cancers and melanoma [32], no effect on EMT was reported. The utilization of multiple molecular and cellular mechanisms to achieve a common biological effect using the same ligand-receptor interaction might be attributed to the complexity of semaphorin/plexin family member interactions and intracellular signaling pathways utilized by these molecules.

Semaphorins and their receptors are recognized as major regulators of cellular morphology and migration involved in various developmental and pathological processes. During embryonic development, they participate in neuronal and non-neuronal cell migration to regulate neural development, heart formation, vasculature formation, and development of the immune system [6]. Specifically, Sema3E/Plexin-D1 signaling regulates endothelial migration involved in heart and vessel formation, and guides the trajectory of descending axonal tracts in developing forebrain [20], [33]–[36]. In the immune system, Sema3E/Plexin-D1 interaction controls migration of positively selected thymocytes into medulla and consequently affects thymic corticomedullary architecture [37]. During tumorigenesis, opposing biological effects of Sema3E/Plexin-D1 signaling have also been reported depending on cellular context. For example, expression of Sema3E in prostate cancers and metastatic melanoma inhibits adhesion and motility of cancer cells [38], [39]. By contrast, Sema3E/Plexin-D1 signaling in colorectal cancers increases cellular invasiveness and metastasis [32]. In addition, although Sema3E in embryonic somites is known to repel Plexin-D1-expressing endothelial cells during vasculogenesis, both anti-angiogenic and pro-angiogenic effects of Sema3E have been reported in cancer cells. For example, down-regulation of Sema3E in melanoma facilitates tumor angiogenesis for distant metastasis [38], while Sema3E overexpression in mammary adenocarcinoma promotes peri-tumor local endothelial cell migration for invasive growth and lung metastasis [15]. The biological effects of Sema3E/Plexin-D1 signaling are clearly diverse and highly dependent on the exact nature of the myriad intracellular signaling pathways available during tumorigenesis. In ovarian endometrial carcinomas, we have provided a previously unknown mechanism of Sema3E/Plexin-D1 signaling that facilitates tumor progression through Snail1-medidated EMT.

Semaphorin/plexin intracellular signaling is highly context-dependent. An emerging picture suggests that semaphorin/plexin interactions trigger crosstalk among multiple intracellular signaling pathways including small GTPases, integrin, MAPK and PI3K/Akt/GSK-3β axes, all of which could lead to cytoskeletal rearrangement and affect cell motility [6], [7], [40]. Consistent with this idea, we show that the cellular EMT induced by Sema3E/Plexin-D1 in ovarian endometrioid carcinoma cells occurs at least partially through PI3K and ERK/MAPK mediated nuclear translocation of Snail1. The involvement of ERK/MAPK activity supports the notion that activation of ERK/MAPK is a hub for multiple semaphorin signaling pathways, as evidenced by previous studies of Sema3A/Plexin-A1, Sema4D/Plexin-B1, and Sema7A signaling [41]–[43]. Activation of ErbB2/Neu kinase is also required for pro-invasive/metastatic activity in tumor cells [32], and we found that ErbB2 transcripts were significantly changed in Sema3E-positive OEC cells in gene profiling via RNA microarray analysis (data not shown). It is therefore likely that Sema3E/Plexin-D1 signaling couples with ErbB2 to regulate MAPK activity in endometrioid cancer cells, as has been shown previously for Sema4D/Plexin-B1 signaling in hipoccampal neurons and HEK293 cells [43]. PI3K has recently been shown to be a main switch for Sema3E/Plexin-D1 signaling in cortical neurons [44]. This switch is regulated by another receptor, Npn-1. Thus, Sema3E/Plexin-D1 signaling normally inhibits PI3K and results in cortical axon repulsion, but at the presence of Npn-1, Plexin-D1 recruits yet another receptor VEGFR2 upon Sema3E binding and then activates PI3K to promote cortical axon attraction and outgrowth. Consistent with this observation, Npn-1 is present in the OEC cells (Fig. 1) and is likely to regulate Sema3E/Plexin-D1 signaling by similar mechanisms. Taken together, our data provide evidence suggesting that PI3K and ERK/MAPK signaling pathways are involved in Sema3E/Plexin-D1-mediated EMT in ovarian endometrioid carcinomas. Further studies are still required to clarify whether other signaling pathways involving integrin, Ras, and Rho family members are involved.

EMT is a strategy frequently exploited by cells for acquisition of increased cell motility either in normal developmental processes or during malignant progression of tumors [25], [26]. Increasing evidence indicates that induction and regulation of Snail family members are critical for EMT to occur [29], [31]. Numerous extracellular signaling molecules including EGF, FGF, HGF, BMPs, WNT, Notch, and TGF-β have been reported to regulate the expression or activity of Snail family members in the context of EMT [31]. Here, we show that Sema3E is capable of acting as an EMT inducer through regulating intracellular Snail1 localization. Consistent with our data, another semaphorin family member in zebra fish, Sema3D, has been shown to regulate the cell cycle of neural crest cells that undergo massive EMT during embryogenesis [45].

Why is Sema3E-mediated EMT so specific to high-grade ovarian endometrioid carcinoma but not other ovarian epithelial tumors? It is well known that human ovarian epithelial cancers arise from the ovarian surface epithelium, which under normal conditions has limited growth potential but can undergo EMT during physiological repair responses [46], [47]. The importance of EMT in normal ovarian physiology is not fully understood, but recent studies have begun to elucidate EMT-associated events in ovarian cancer development and progression. Factors such as endothelin A receptor/endothelin-1 axis, autocrine BMP4 signaling pathway, and 17ß-estradiol have been reported to trigger EMT and promote tumor progression by up-regulating Snail1 activity in human ovarian cancer cells [48]–[50]. The diversity of EMT-triggers in various tissue types of ovarian epithelial cancers, including the Sema3E signaling pathway reported here, suggests the existence of sub-populations of ovarian surface epithelium that respond differentially toward growth factors and cytokines under normal conditions, and may underline their specific susceptibilities to different factors for malignant transformation and tumor progression.

## Materials and Methods

### Case selection

A total of 40 cases diagnosed as ovarian endometrioid carcinoma were identified after searching the pathological records from January 1999 to December 2005 registered in National Taiwan University Hospital (NTUH), Taipei, Taiwan. Additional 40 cases each for ovarian clear cell carcinoma, serous carcinoma and mucinous carcinoma were also included for immunohistochemistry studies. Tissue blocks were derived from the archives of the Department of Pathology, NTUH, following the guidelines set forth by Tissue Committee at NTUH. Selected demographic information including age, sex, histological subtype and grade, and outcome of survival for each patient included in this study was retrieved from the hospital cancer registry as documented [18].

### Antibodies and Chemicals

Rabbit polyclonal antisera against Sema3E or Plexin-D1 were generated by immunizing rabbits with KLH-conjugated synthetic peptides that correspond to a.a. (amino acids) 67–84 of human Sema3E (NM_012431) or a.a. 191–204 of human Plexin-D1 (NM_015103), respectively (Sigma). The polyclonal antibodies were purified by affinity chromatography (CNBr-activated Sepharose 4B beads, Amersham), and the antibody specificity was examined by Western blotting and by tissue-immunohistochemistry (Fig. S1A, B, C). Other antibodies used include antibodies to Snail1, Snail2 (Slug), GFP, ERK1+ERK2 (Abcam), phospho-ERK1+ERK2 (Santa Cruz), Vimentin, E-Cadherin, Fibronectin (BD Biosciences), Cytokeratin, œ-Tubulin (Oncogene), Myc (Invitrogen), and phospho-Akt(T308), phospho-Akt(S473), Furin (R&D Systems). Chemicals used include Leptomycin B (Sigma), Furin inhibitor I (Decanoyl-RVKR-CMK) (Calbiochem), GF109203X, LY294002, Wortmannin (Merck), SB203580, SP600125, PD98059 (Biosource).

### Plasmid constructs

The full-length human Sema3E cDNA was purchased from ATCC and was subcloned into either a pCMVtag, a pcDNA3.1/Myc-His, or a pEGFP vector by conventional cloning technique. The p61-Sema3E isoform (p61-Sema3E-Flag), which corresponds to a.a. 32–560 of the full-length Sema3E, was PCR-generated and subcloned in frame into the EcoRI and SalI sites of pCMVtag-2A. Step-by-step site-directed mutagenesis was used to generate pSema3E-p87(R557S, R559G, R560G)-GFP by synthesizing primers flanking the designed mutation sites and following the manufacture's instructions (Stratagene). Human Plexin-D1, Snail1, and Snail2 (Slug) cDNA constructs were generated by PCR from human fetal cDNA library (Clontech) and subcloned into the mammalian expression vectors. Plexin-D1-specific small interfering RNA (siRNA-1: 5′-TTTGAGCAGGTGGTGGCT-3′, nucleotide (nt) 5718-5736 from NM_015103, CDS; siRNA-2: 5′-GGACTCGCTGAGCGTGCGGG-3′, nt.4801-4820), Sema3E-specific siRNA (siRNA-1: 5′-AAGTATATTTCTTTTTTA-3′, nt.727-745 from NM_012431, CDS; siRNA-2: 5′-TTCCAAACCTGAGCATTACC-3′, nt.2900-2920), and a nonspecific duplex oligo (as a negative control) were synthesized and cloned into pSUPER plasmid (OligoEngine). pLKO.1-shSnail1 plasmids targeting five different sequences (NM_005985, CDS: nt.757-777; nt.514-534; nt.504-524; nt.136-156; nt.108-128) and pLKO.1-shSlug plasmids targeting four different sequences (NM_003068, CDS: nt.1315-1335; nt.810-830; nt.865-885; nt.254-274) were purchased and prepared from National RNAi Core facility, Academia Sinica, Taiwan.

### Cells lines, lentiviral infection, and plasmid transfection

The parental and established OEC stable cell lines were maintained in complete media (DMEM plus 10% FBS and 1% penicillin/streptomycin), with or without 500 µg/ml G418 at 37°C incubator inflated with 5% CO_2_. The plasmid was introduced by lipofectAMINE2000 (Invitrogen). Cells were infected with lentiviral preparation with MOI:120 plus 8 mg/ml polybrene (Sigma).

### Measurement of cell proliferation and viability

Cell proliferation curves were determined by counting viable cells with trypan blue exclusion. Cell viability was determined from the sub-G1 fraction in the flow cytometry by using propidium iodide (Molecular Probes).

### Semi-quantitative RT-PCR

Total cellular RNA from tissue or cells was isolated using MaestroZol™ kit (MAESTROGEN), according to the manufacturer's instructions. Then, cDNA was synthesized from total RNA (5 µg) with oligo d(T) and SuperScript II reverse transcriptase (Invitrogen). The primer sets used were: Sema3A (Genbank accession number: NM_006080, nt.902-921, nt.1346-1328 and nt.1929-1951, nt.2052-2032 for real-time PCR); Sema3B (NM_001005914, nt.178-193, nt.775-755; nt.1489-1507, nt.1603-1584 for real-time PCR); Sema3C (NM_006379, nt.563-582, nt.1325-1304; nt.569-588, nt.688-665 for real-time PCR); Sema3D (NM_152754, nt.2059-2076, nt.2373-2355); Sema3E (NM_012431, nt.1475-1495, nt.2031-2011; nt.1633-1658, nt.1754-1731 for real-time PCR); Sema3F (NM_004186, nt.781-799, nt.909-892); Npn1 (NM_003873, nt.524-541, nt.1184-1174; nt.591-615, nt.704-685 for real-time PCR); Npn2 (NM_201267, nt.796-812, nt.1449-1429); Plexin-A1 (NM_032242, nt.2519-2536, nt.3052-3034; nt.7737-7759, nt.7879-7858 for real-time PCR); Plexin-A2 (NM_025179, nt.4172-4190, nt.4736-4717); Plexin-A3 (NM_017514, nt.2788-2805, nt.3102-3095); Plexin-B1 (NM_002673, nt.4285-4302, nt.4640-4622); Plexin-C1(NM_005761, nt.3273-3291, nt.3865-3847); Plexin-D1 (NM_015103, nt.4017-4034, nt.4474-4456; nt.5018-5028, nt.5132-5108 for real-time PCR). The PCR analyses were performed in two methods: (1) PCR analysis by gel electrophoresis: The PCR amplification was done as follows: denaturing at 94°C for 30 s, annealing at 55°C for 30 s, and extension at 72°C for 45 s. The PCR products were resolved in gel electrophoresis. The number of cycles was determined by pilot experiments so that all amplifications took place within the linear range. Parallel RT-PCR experiments were conducted using the housekeeping gene *GAPDH* as an internal standard and all expression values were expressed as a ratio of GAPDH levels. (2) PCR in real time: The real-time PCR was performed using ABI PRISM® 7900HT Sequence Detection System in the presence of the SYBR® Green Realtime PCR Master Mix (TOYOBO). Each amplified sample was analyzed for homogeneity using dissociation curve analysis. The relative expression value of each gene to GAPDH was calculated using the SDS software v2.2 (Applied Biosystems). Each reverse transcription and PCR assay was performed at least in triplicate.

### RNA *in situ* hybridization

RNA *in situ* hybridization with indicated DIG-labeled riboprobes was performed in tissue sections and cells as previously described [51].

### Wound-healing, 3D-matrigel invasive growth, and transwell migration assays

For the wound-healing assay, cultured cells were wounded, photographed each hour, and quantified as the average linear migratory speed of the wound edges over 12∼16 hours. The 3D-matrigel invasion assay was done by placing a solidified cell cake composed of aggregates of cells inside a 30 µl-volume of growth-factor reduced Matrigel (Becton Dickinson) in one well of 4-well plates covered with complete medium. After 48∼60 hours, radial migration distance from central cell aggregates was photographed and measured in at least triplicate experiments. Cell invasion assay was performed in Boyden chambers containing polycarbonate filters with 8 µm pore size and pre-coated with Matrigel (Costar). Cells were placed in the upper wells of 24-chambers at a density of 5×10^4^/100 µl complete medium for 48 hours, and those cells adherent to the bottom surface of the filter were fixed, stained with crystal violet, and counted by light microscopy.

### Western blotting, immunohistochemistry, time-lapsed and confocal immunofluorescence microscopy

Western blot and immunohistochemistry and confocal microscopy were performed as described [51]. For numerical counts of Snail1 subcellular localization, images were acquired with Leica confocal microscopy and at least 400 cells in randomly selected high-power field (400×) were counted for each experiment. For time-lapsed recording, cells grown on 6-well plates were enclosed in a chamber designed for continuous video recording. In some assays, recombinant Sema3E (R&D) was added to P0 cells. Effective optimal concentration (2 nM) was experimentally titrated and determined. Images were recorded once for every 5 mins with Leica DMIRB microscope equipped with Photometrics CoolSNAP of CCD. Fluorescent intensity and migration velocity were measured by Metamorph version 7.5.6.

### Luciferase assay

Human E-cadherin promoter (−178/+92) was cloned into the pGL3-basic vector (Promega). Site-directed mutagenesis was used to generate pGL3-E-cad-promoter (−178/+92) with mutated E-box (−69/−64CAGGTG, −18/−13 CACCTG, and +32/+37CACCTG4 mutated as AACCTA) following the manufacture's instruction (Stratagene). Cells were seeded in 24-well plates at the density of 3×10^4^ cells/well, and were co-transfected with pGL3-control, or pGL3-basic, or pGL3-E-cadherin promoter plus pRL-TK per well. Cell lysate was collected after 48 or 72 hours of transfection. Expression of the firefly and Renilla luciferase was assayed in accordance with Dual-Luciferase® Reporter Assay System (Promega).

### Experimental lung metastasis

The OEC cells (2×10^5^) in 200 µl of PBS were injected into the tail vein of female NOD/SCID mice (age: 6–8 weeks). Mice were sacrificed between days 35∼56 after injection and the organ were formalin-fixed and subjected for morphology and immunohistochemistry analysis.

## Supporting Information

Figure S1 **A, B. Generation and validation of anti-human Sema3E and human Plexin-D1 antibodies.** A polyclonal antiserum against human Sema3E, Sema3E-O4, was generated (A). This antiserum (1∶500 dilution) recognizes Sema3E transiently expressed in COS cells exactly at the same band detected by a commercially purchased anti-Sema3E antibody, Sema3E-T19. The immuno-intensity detected by Sema3E-O4 is diminished in the presence of Sema3E-RNAi. Note that the size of the band recognized by Sema3E-O4 and Sema3E-T19 indicates the p61-Sema3E isoform fused to EGFP, suggesting cleavage of the transfected full-length Sema3E (p87) in the COS cells. Besides, cells transiently transfected with the vector p-Sema3Ep87(R557S, R559G, R560G)-GFP expresses the un-cleaved Sema3E-p87 fused to GFP. Asterisk: non-specific band detected by Sema3E-O4. Arrowhead: non-specific band detected by Sema3E-T19. In (B), the polyclonal anti-Plexin-D1 antisera specifically recognize Myc-tagged Plexin-D1 expressed in COS cells by immunoblot. **C**. Sema3E immunohistochemistry in human tissue arrays. Anti-Sema3E antisera, not the pre-immune serum, recognize human tumor cells in lung adenocarcinoma and invasive ductal adenocarcinoma of the breast as reported before [15]. Normal lung tissue and other epithelial cancers examined here such as adenoid cystic carcinoma of salivary gland and renal cell carcinoma (clear cell type) did not express Sema3E.
(TIF)

Figure S2 **A. RNA** ***in situ*** **hybridization reveals more Sema3E, less Sema3F, and comparable Plexin-D1 and Npn1 transcripts in P4 cells than in P0 cells.** Sense-control for each probe was shown in the rectangle of the left lower corner. Scale bar, 10 µm. **B**. mRNA expression of class 3 semaphorin, Npn and plexin in P4 cells relative to P0 cells (shown by fold-change) as revealed by real-time PCR. **C**. All constructed OEC cell lines involving Sema3E/Plexin-D1 signaling activity shows similar sub-G1 fraction and pattern of cell cycle progression in flow cytometry using propidium iodide stain. **D**. Similar cell proliferation curves are observed in all constructed OEC lines as determined by trypan blue exclusion. **E**. Representative photographs of OEC cell aggregates with varying Sema3E/Plexin-D1 activity in 3D-Matrigel photographed after 63-h culture. P0-3E-_1_ cells migrate farther than P0 and P0-3E-_10_ cells, whereas RNAi-knockdown of Plexin-D1 in P0-3E-_1_ cells (P0-3E-_1_/siD1) significantly reduces the migration distance. The migratory ability of P4 cells is also diminished by RNAi-depletion of Sema3E (P4/si3E).
(TIF)

Figure S3 **Furin-processed p61-Sema3E is required for promoting the** ***in vitro*** **migratory/invasive ability of OEC cells conferred by Sema3E.** **A**. Furin is present in all constructed OEC clones as evident by immunoblotting. **B**. The furin inhibitor, decanoyl-RVKR-chloromethylketone, prevents the full-sized p87-Sema3E from cleavage into p61 isoform in P0-3E-_1_ cells. **C**, **D**. P0 cells that stably expressed only the p61 isoform (P0-3Ep61-_1_ and P0-3Ep61-_2_ cell lines) exhibit similar migration-promoting effect comparable to P0-3E-_1_ cells in wound-healing process. Scale bar, 0.5 mm; D, n = 12, **, *P*<0.005, paired *t*-test. **E**, **F**, **G**. Exogenous p61-Sema3E promotes migration of Sema3E-negative-P0 cells in a dose-dependent manner. Representative images in (E) and bar graph in (F)(n = 5) shows that p61-Sema3E-conditioned media (Sema3E-p61-CM) accelerates P0 cell migration rate in the wound-healing process, which is concentration-dependently attenuated when the Sema3E-p61-CM is serially-diluted with mock-transfected conditioned media (Mock-CM) as evident by Western blotting (G). The migration-promoting effect is specifically blocked when the Sema3E-p61-CM is pretreated with an anti-Sema3E antibody (anti-Sema3E), but not with a non-specific antibody (anti-IgG Ab). Scale bar, 0.5 mm, **, *P*<0.005; *, *P*<0.05, paired *t*-test.
(TIF)

Figure S4 **A**. **Immunofluorescence of E-cadherin performed in confluent cells shows membranous (cell border) and cytoplasmic staining in P0 cells, but the staining in P0-3E-_1_ cells (upper panels) is greatly reduced.** By contrast, vimentin-immunoreactivity is detected in P0-3E-_1_ cells, but not in P0 cells (lower panels). Scale bar, 20 µm. **B**, **C**, **D**. Time-lapse tracing of the sub-cellular localization of GFP-tagged Snail1 protein transfected in high-Sema3E expressing P0-3E-_1_ cells. Shown here is the result from a 2-hour recording with 5-min time-lapse intervals. Note that nuclear localization of Snail1 correlates with spindle morphology of the cell (B). The decrease in the nuclear fluorescent intensity of Snail1 (C) correlates well with slower cell motility (D).
(TIF)
