# Supplementary figures and images for: Sema3E/Plexin-D1 Mediated Epithelial-to-Mesenchymal Transition in Ovarian Endometrioid Cancer
Source: PLoS One. 2011 Apr 29;6(4):e19396. doi: 10.1371/journal.pone.0019396 (PMC3084850; doi:10.1371/journal.pone.0019396)

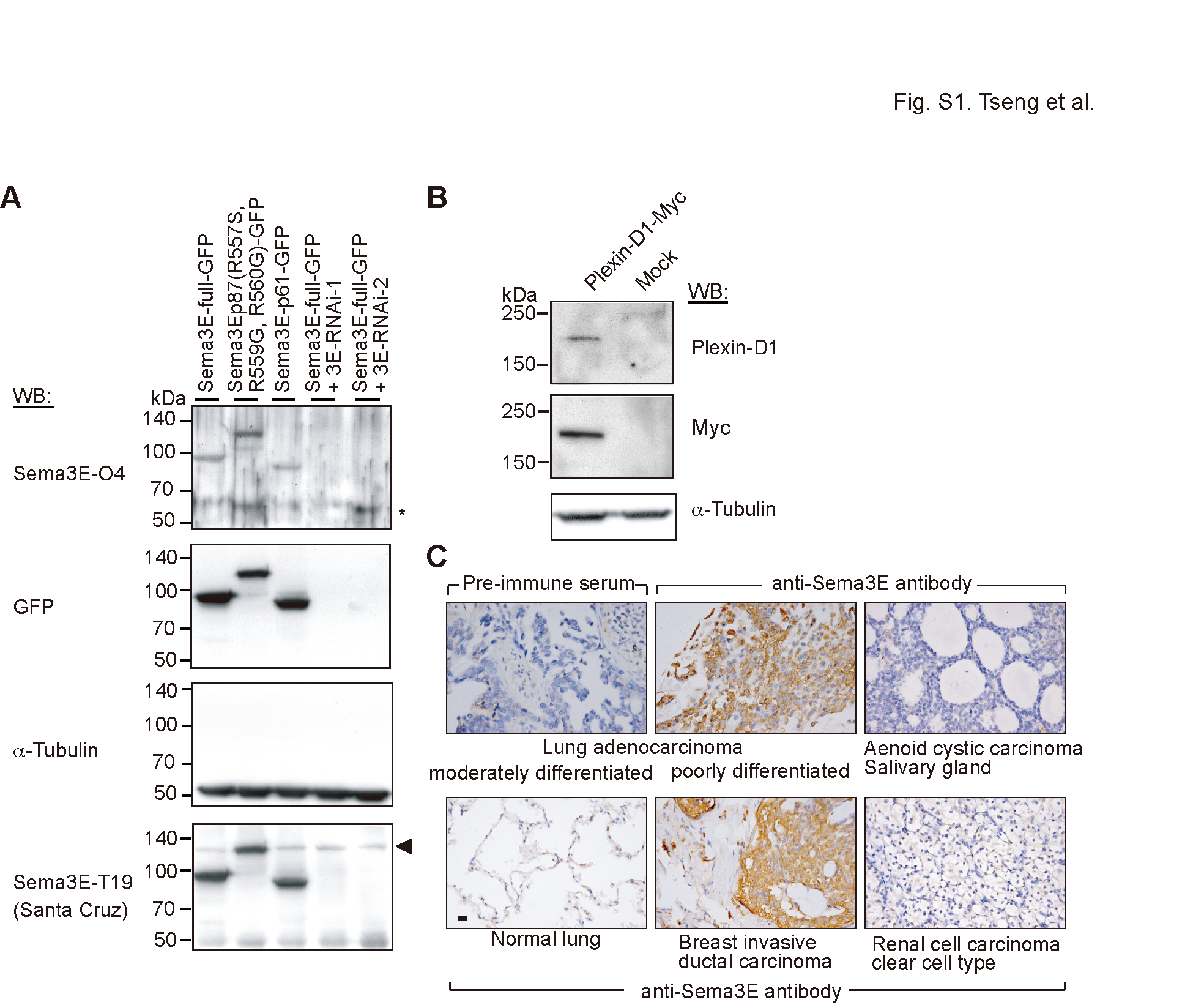

Supplement: Figure S1 — A, B. Generation and validation of anti-human Sema3E and human Plexin-D1 antibodies. A polyclonal antiserum against human Sema3E, Sema3E-O4, was generated (A). This antiserum (1∶500 dilution) recognizes Sema3E transiently expressed in COS cells exactly at the same band detected by a commercially purchased anti-Sema3E antibody, Sema3E-T19. The immuno-intensity detected by Sema3E-O4 is diminished in the presence of Sema3E-RNAi. Note that the size of the band recognized by Sema3E-O4 and Sema3E-T19 indicates the p61-Sema3E isoform fused to EGFP, suggesting cleavage of the transfected full-length Sema3E (p87) in the COS cells. Besides, cells transiently transfected with the vector p-Sema3Ep87(R557S, R559G, R560G)-GFP expresses the un-cleaved Sema3E-p87 fused to GFP. Asterisk: non-specific band detected by Sema3E-O4. Arrowhead: non-specific band detected by Sema3E-T19. In (B), the polyclonal anti-Plexin-D1 antisera specifically recognize Myc-tagged Plexin-D1 expressed in COS cells by immunoblot. C. Sema3E immunohistochemistry in human tissue arrays. Anti-Sema3E antisera, not the pre-immune serum, recognize human tumor cells in lung adenocarcinoma and invasive ductal adenocarcinoma of the breast as reported before [15]. Normal lung tissue and other epithelial cancers examined here such as adenoid cystic carcinoma of salivary gland and renal cell carcinoma (clear cell type) did not express Sema3E. (TIF) [file pone.0019396.s001.tif]

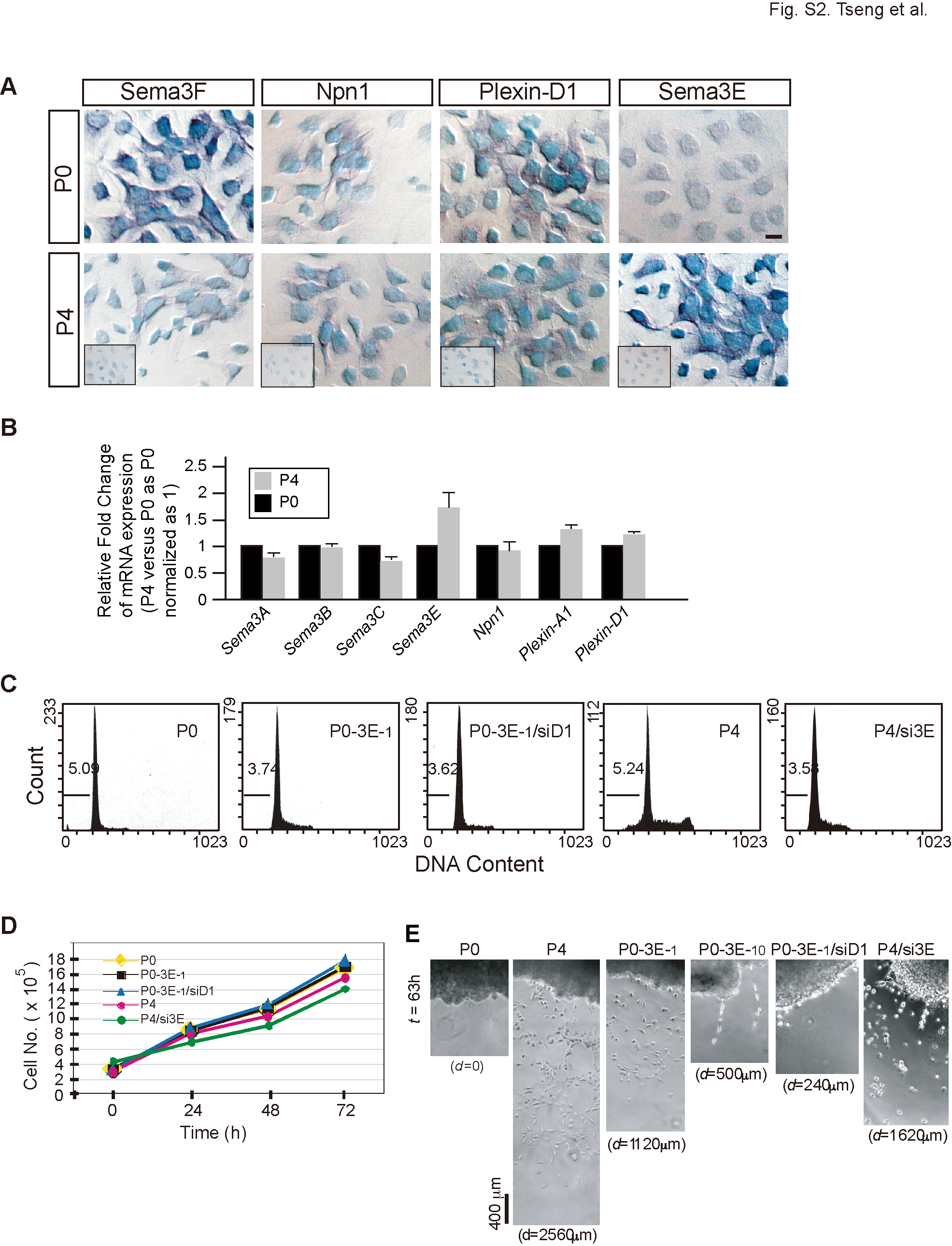

Supplement: Figure S2 — A. RNA in situ hybridization reveals more Sema3E, less Sema3F, and comparable Plexin-D1 and Npn1 transcripts in P4 cells than in P0 cells. Sense-control for each probe was shown in the rectangle of the left lower corner. Scale bar, 10 µm. B. mRNA expression of class 3 semaphorin, Npn and plexin in P4 cells relative to P0 cells (shown by fold-change) as revealed by real-time PCR. C. All constructed OEC cell lines involving Sema3E/Plexin-D1 signaling activity shows similar sub-G1 fraction and pattern of cell cycle progression in flow cytometry using propidium iodide stain. D. Similar cell proliferation curves are observed in all constructed OEC lines as determined by trypan blue exclusion. E. Representative photographs of OEC cell aggregates with varying Sema3E/Plexin-D1 activity in 3D-Matrigel photographed after 63-h culture. P0-3E-1 cells migrate farther than P0 and P0-3E-10 cells, whereas RNAi-knockdown of Plexin-D1 in P0-3E-1 cells (P0-3E-1/siD1) significantly reduces the migration distance. The migratory ability of P4 cells is also diminished by RNAi-depletion of Sema3E (P4/si3E). (TIF) [file pone.0019396.s002.tif]

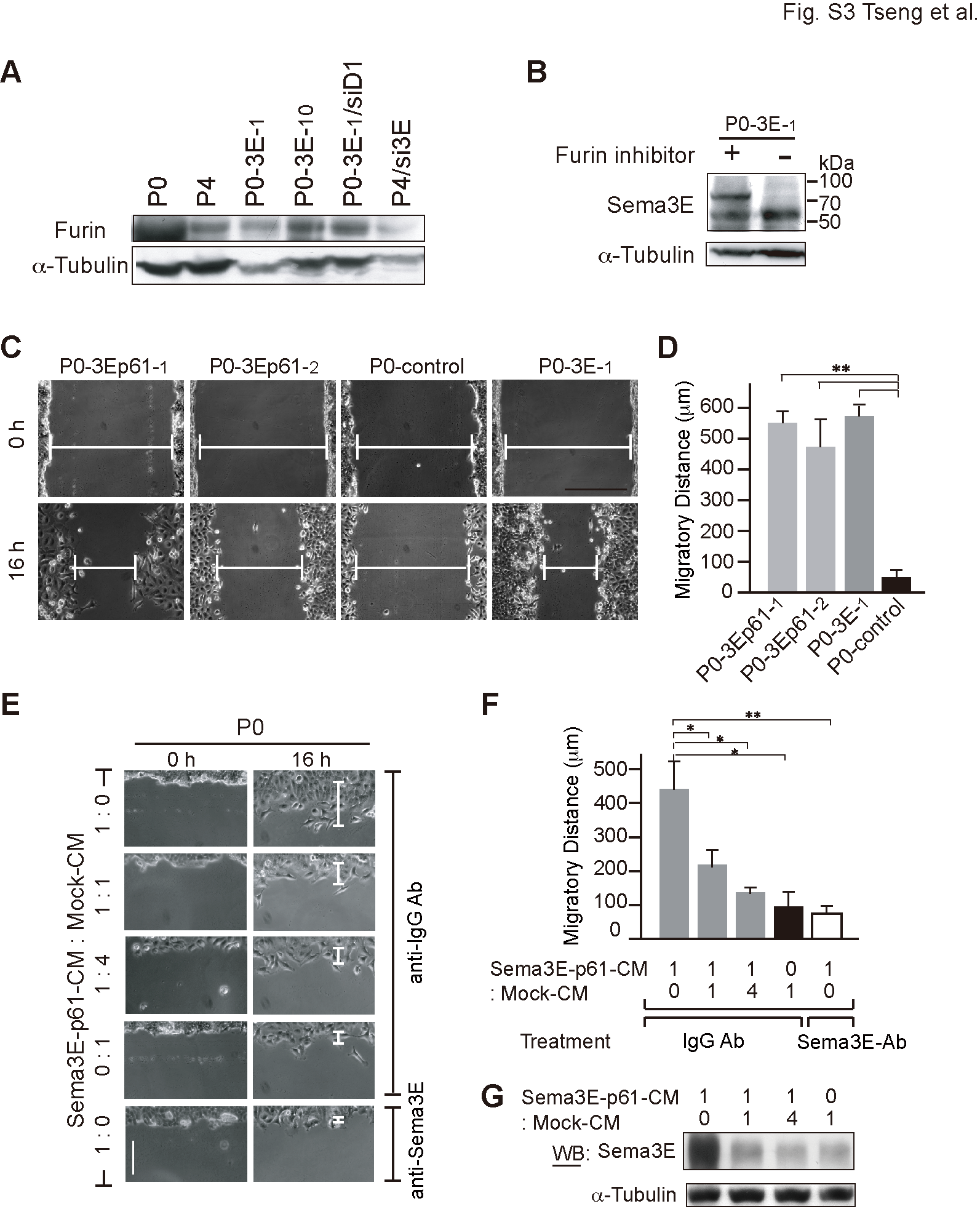

Supplement: Figure S3 — Furin-processed p61-Sema3E is required for promoting the in vitro migratory/invasive ability of OEC cells conferred by Sema3E. A. Furin is present in all constructed OEC clones as evident by immunoblotting. B. The furin inhibitor, decanoyl-RVKR-chloromethylketone, prevents the full-sized p87-Sema3E from cleavage into p61 isoform in P0-3E-1 cells. C, D. P0 cells that stably expressed only the p61 isoform (P0-3Ep61-1 and P0-3Ep61-2 cell lines) exhibit similar migration-promoting effect comparable to P0-3E-1 cells in wound-healing process. Scale bar, 0.5 mm; D, n = 12, **, P<0.005, paired t-test. E, F, G. Exogenous p61-Sema3E promotes migration of Sema3E-negative-P0 cells in a dose-dependent manner. Representative images in (E) and bar graph in (F)(n = 5) shows that p61-Sema3E-conditioned media (Sema3E-p61-CM) accelerates P0 cell migration rate in the wound-healing process, which is concentration-dependently attenuated when the Sema3E-p61-CM is serially-diluted with mock-transfected conditioned media (Mock-CM) as evident by Western blotting (G). The migration-promoting effect is specifically blocked when the Sema3E-p61-CM is pretreated with an anti-Sema3E antibody (anti-Sema3E), but not with a non-specific antibody (anti-IgG Ab). Scale bar, 0.5 mm, **, P<0.005; *, P<0.05, paired t-test. (TIF) [file pone.0019396.s003.tif]

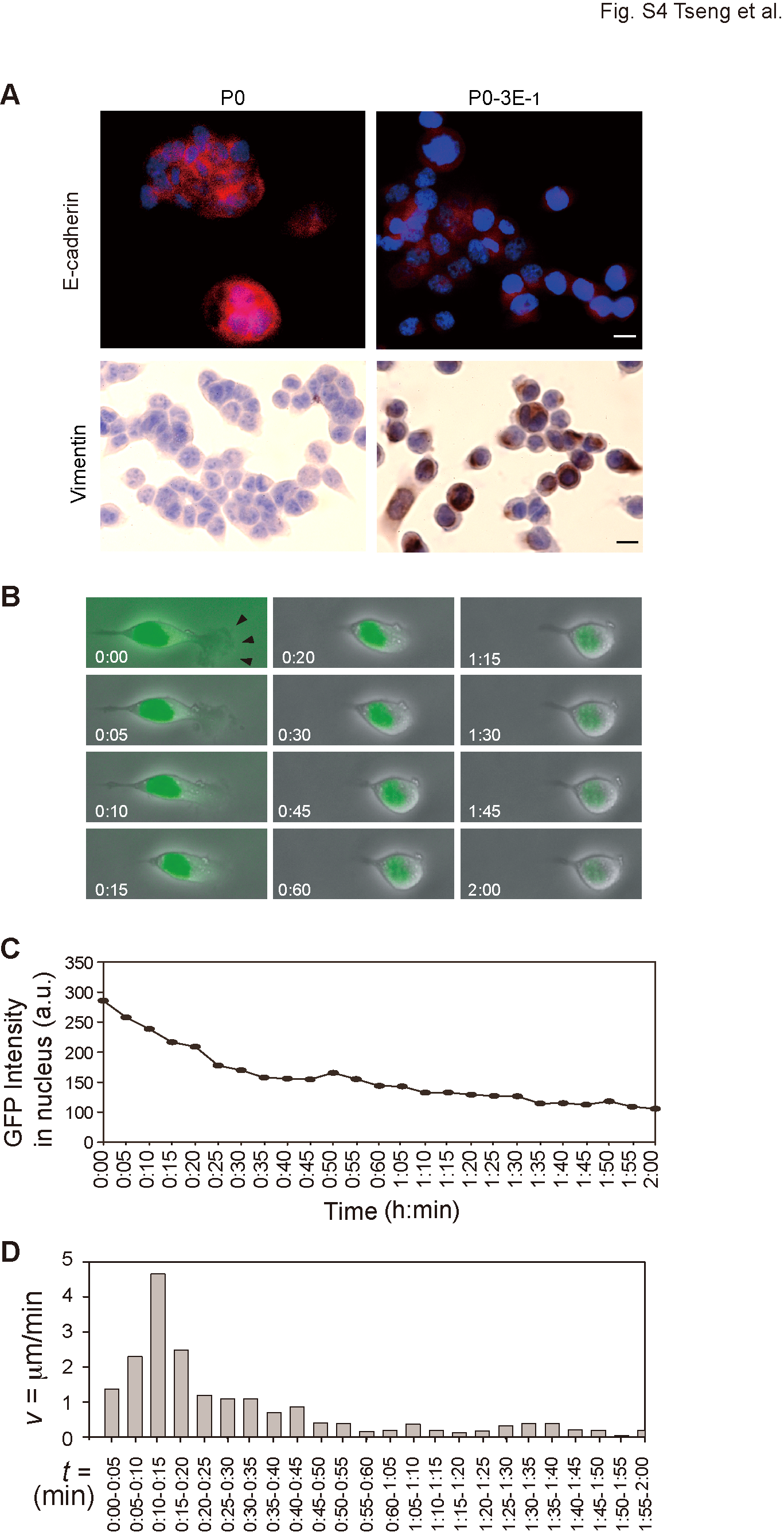

Supplement: Figure S4 — A. Immunofluorescence of E-cadherin performed in confluent cells shows membranous (cell border) and cytoplasmic staining in P0 cells, but the staining in P0-3E-1 cells (upper panels) is greatly reduced. By contrast, vimentin-immunoreactivity is detected in P0-3E-1 cells, but not in P0 cells (lower panels). Scale bar, 20 µm. B, C, D. Time-lapse tracing of the sub-cellular localization of GFP-tagged Snail1 protein transfected in high-Sema3E expressing P0-3E-1 cells. Shown here is the result from a 2-hour recording with 5-min time-lapse intervals. Note that nuclear localization of Snail1 correlates with spindle morphology of the cell (B). The decrease in the nuclear fluorescent intensity of Snail1 (C) correlates well with slower cell motility (D). (TIF) [file pone.0019396.s004.tif]
